# The protandric life history of the Northern spot shrimp *Pandalus platyceros*: molecular insights and implications for fishery management

**DOI:** 10.1038/s41598-020-58262-6

**Published:** 2020-01-28

**Authors:** Tom Levy, Sherry L. Tamone, Rivka Manor, Esther D. Bower, Amir Sagi

**Affiliations:** 1 Department of Life Sciences, Ben-Gurion University of the Negev, P.O. Box 653, Beer Sheva, 84105 Israel; 2 University of Alaska Southeast, 11066 Auke Lake Way Hwy, Juneau, AK 99801 USA; 3 The National Institute for Biotechnology in the Negev, Ben-Gurion University of the Negev, P.O. Box 653, Beer Sheva, 84105 Israel

**Keywords:** Reproductive biology, Animal physiology

## Abstract

The Northern spot shrimp, *Pandalus platyceros*, a protandric hermaphrodite of commercial importance in North America, is the primary target species for shrimp fisheries within Southeast Alaska. Fishery data obtained from the Alaska Department of Fish and Game indicate that spot shrimp populations have been declining significantly over the past 25 years. We collected spot shrimps in Southeast Alaska and measured reproductive-related morphological, gonadal and molecular changes during the entire life history. The *appendix masculina*, a major sexual morphological indicator, is indicative of the reproductive phase of the animal, lengthening during maturation from juvenile to the male phase and then gradually shortening throughout the transitional stages until its complete disappearance upon transformation to a female. This morphological change occurs in parallel with the degeneration of testicular tissue in the ovotestis and enhanced ovarian vitellogenesis. Moreover, we obtained the entire mRNA sequence of the yolk protein precursor, vitellogenin, and monitored its transcript levels throughout the entire shrimp life-cycle. Vitellogenin transcript levels in the hepatopancreas increased in the early transitional stage until reaching a peak prior to extruding eggs. Such transcriptomic analyses, coupled with a comprehensive description of the gonad, external sex characters and timing of the reproductive life history of spot shrimps contribute to a better understanding of the hermaphroditic reproduction process in the cold Southeast Alaskan waters. This knowledge can contribute to a revision of current conservation efforts to maintain wild populations sustainable for both commercial and ecological considerations.

## Introduction

The Northern spot shrimp, *Pandalus platyceros*, is the largest species in the family Pandalidae with sizes reaching up to 61 mm carapace length^1^. It is widely distributed in the eastern North Pacific Ocean, from Alaska and British Columbia, along the West coast to as far south as San Diego, CA, and within the western North Pacific Ocean along the Siberian east coast from the Sea of Japan to Korea Strait^2^. During its life history, *P. platyceros* inhabits waters from the shallow intertidal to depths of more than 500 m^2^. *P. platyceros* is the primary target species for a shrimp pot fishery in Southeast Alaska. Since 1970 there has been a progressive increase in the commercial harvest of *P. platyceros* within Southeast Alaska from 9,700 Kg harvested in the 1970’s to 124,500 Kg harvested in the 1980s, 396,000 harvested throughout the 1990s and 417,000 Kg in the 2000s^3^. From the 1990’s, shrimp populations have been declining and consequently, careful management of spot shrimp populations including strict fishing regulations were imposed. Populations of Northern spot shrimp in Southeast Alaska have not recovered to their original numbers. Fishing regulations are developed not only for the management of pandalid shrimps in Southeast Alaska^3^, but also for penaeid shrimps in Australia^4^, and different fish in Kenya^5^, Faroe Islands^6^, Florida and St. Lucia^7^. Such regulations include technical limitations such as mesh size, restrictions on fishing gear, temporal closures and even marine reserves^3–7^. While target-species specific regulations are designed to sustain the species’ populations, other fishing practices may increase fishing mortality. Consequently, bycatch of non-target species can cause significant ecological implications such as shifts in population demographics which affect interactions between ecosystem components^8^. Specific regulations imposed on the *P. platyceros* fishery include mesh restrictions which allow for the escapement of shrimps below a certain size, seasonal fishing restrictions which prevent the harvest of females during the egg hatching period, and harvest restrictions which use fixed annual quotas in designated sites that are adjusted based upon survey of shrimp populations^3^. Understanding the complete life history of the protandric Northern spot shrimp is therefore essential to effectively inform important fishery regulations.

Similar to other protandric hermaphrodite shrimps, *P. platyceros*^9^ begins its adult life as a male with hermaphrodite gonads (ovotestis) in which the testicular component is functional. Later the ovotestis transforms into a functional ovary^10^. The time required to complete the transformation from male to female may differ across latitudes^11^.

The reproductive cycle of the spot shrimp includes mating, brooding, hatching embryos and molting prior to the next mating. Spot shrimps mate during late summer after embryonic hatching and after a female has undergone the pre-mating molt (PMM), in which it renews its hair-like ovigerous setae where the extruded eggs are attached and brooded^12^. Eggs are extruded in the fall and larvae hatch in the spring^2^. The post-larval stage occurs approximately 40 days post-hatching and then the benthic juvenile period begins until juveniles matures into the male phase^11,13^. Males may mate multiple times and will go through a transitional period prior to maturing as females^14–16^.

Laboratory studies have been valuable in describing the life history of the spot shrimp. As with other shrimp species, the reproductive phase of the animal can be visually determined by examining structures on the proximal part of the second pleopod’s endopod. While the *appendix interna* (*AI*) is present in both males and females, the *appendix masculina* (*AM*) is a typical male secondary sex character^17,18^. An increase in the *AM*/*AI* ratio was produced in a gonochoristic female prawn that was manipulated through an induced sex-reversal to a male and thus developed an *AM*^19^. Alternatively, the *AM* of males that were experimentally manipulated and sexually reversed into females regressed^20^. In *P. platyceros*, the *AM* develops as the juvenile shrimp matures to a male, and it naturally decreases in length through the transitional molt stages until it disappears altogether as the shrimp fully transforms into a female^21^. Therefore, examination of the *AM/AI* ratio serves as a noninvasive method to determine the sexual stage of a given shrimp.

The physiological transformation of a male into a female can be followed by monitoring the expression pattern of the gene encoding vitellogenin (Vg) reflecting the feminine physiological reproductive state in spot shrimps. Vg is a precursor of the major yolk protein vitellin, which is found in the eggs of most oviparous animals including crustaceans^22,23^. During vitellogenesis, Vg is synthesized in the hepatopancreas, secreted to the circulatory system and then transported to the ovary, where it is processed and accumulated as vitellin^24–26^. However, in some species Vg is produced not only in the hepatopancreas, but also in the ovary itself^27^. Vitellogenesis and oocyte maturation are well described processes in females of gonochoristic shrimps and prawns^28–30^, as well as protandric shrimps^25^. However, measuring *Vg* transcript levels during the entire life-cycle of a sequential hermaphrodite shrimp, from the undifferentiated juvenile to the female phase, has not been previously described.

In this study we analyzed harvest data obtained from the spot shrimp fishery in Southeast Alaska over the last two decades, in order to demonstrate the declining trends of the fishery in this region. Within a district of this region of Southeast Alaska, we collected specimens of the different reproductive phases of the entire life history of *P. platyceros*. We used morphological indicators combined with molecular and transcriptomic techniques to study spot shrimp reproductive physiology in order to devise a temporal model of reproductive events. Understanding the complete life history of the spot shrimp could contribute to better conservation efforts and support more sustainable fishery management practices. This understanding could also lead to new approaches for the mariculture of Northern spot shrimps.

## Results

### GHL and actual harvest levels of *P. platyceros* in Southeast Alaska

**Figure 1:**
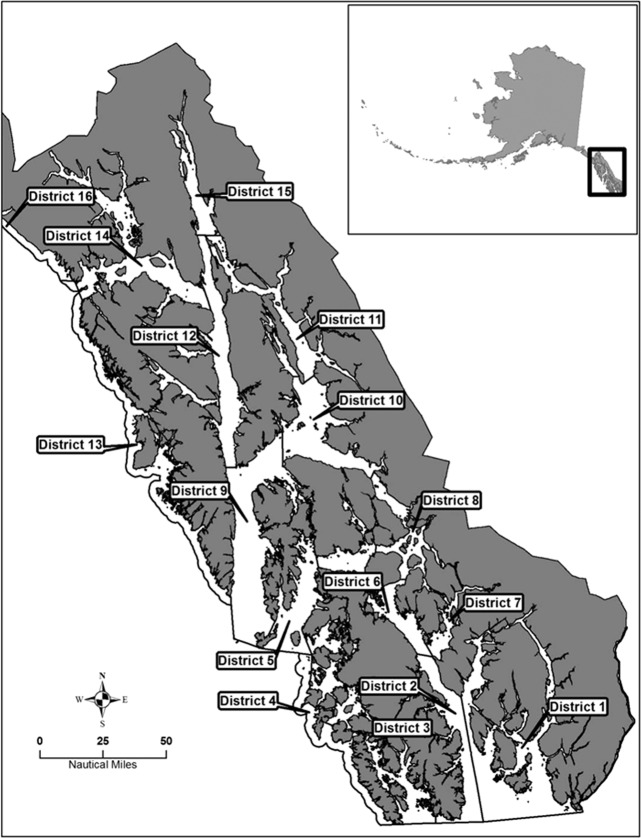
Shrimp pot fishery management sites in Southeast Alaska. The area is divided to 16 units. Image adapted from the Alaska Department of Fish and Game.

**Figure 2:**
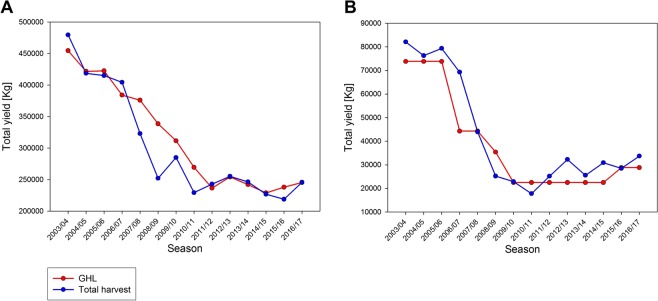
Spot shrimp fishery data (2013–2017). (**A**) Total guideline harvest levels (GHL; red) and total harvest (blue) in the 12 surveyed sites. (**B**) Guideline harvest levels (GHL; red) and total harvest (blue) in the sampling area of the present study (district 1 - see Fig. 1).

Based on population size assessments from 2003 to 2012, total GHL (Guideline Harvest Levels) for the 12 survey sites (Fig. 1; excluding 4, 5, 14 and 16) were reduced by approximately 50% from 454,500 Kg of shrimps in 2003 to 236,520 Kg in 2012 (Fig. 2A). From 2012 to 2017 the GHL remained constantly reduced. Not each harvest district experienced the same decrease in GHL or harvest and in fact, the GHL in the sampling area from which we obtained our shrimps (district 1), was reduced by 70% from 73,800 Kg of shrimps in 2003 to 22,500 Kg in 2009 and has been maintained at a reduced level through 2017 (Fig. 2B). The actual harvest of the fishery correlated with the designated GHL in Southeast Alaska. Between 1993 and 2007 the total harvest in the 12 sites assessed for this study (excluding districts 4, 5, 14 and 16; Fig. 1) fluctuated between 373,500–495,000 Kg. In 2008, fishermen harvested 252,045 Kg across all districts, since 2008, harvest of spot shrimps has remained depressed with an average harvest of 244,620 Kg (SD = 19.44 Kg). The total harvest of spot shrimps in district 1 (the district in which we sampled our shrimps for this study), was 64,215–82,080 Kg between 1993 and 2007, and then decreased to 17,775–33,705 Kg between 2008 and 2017.

### AM and AI in P. platyceros

**Figure 3:**
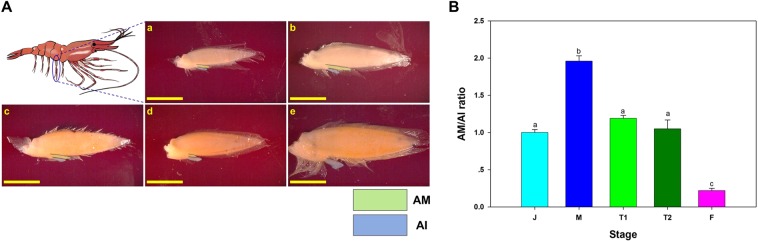
Sex-related morphological characters in *P. platyceros*. (**A**) Second pleopods at different reproductive stages. The location of the second pleopod is shown in the blue circle (top left). *Appendix masculina* (*AM*; green) and *appendix interna* (*AI*; blue) are highlighted in (a) Juvenile, (b) Male, (c) Early transitional, (d) Late transitional and (e) Female. Bar scales (=5 mm) are consistent in all photos to highlight the pleopod’s size difference between different stages. (**B**) *AM/AI* ratio in different reproductive stages. (J) Juvenile, (M) Male, (T1) Early transitional, (T2) Late transitional and (F) Female. Error bars represent standard error of the means and statistical difference is indicated with letters.

The *AM* is one of the most well defined external characteristics of male shrimps^17^, and indeed, while the size of the *AI* remained almost constant, the *AM* extended as the animal entered the male phase, while becoming shorter during the transformation phase into female (Fig. 3A). Consequently, the *AM/AI* ratio increases from the juvenile to the male stage and then decreases during the transformation period to the female stage. The average ± SE of the *AM/AI* ratio is 1.00 ± 0.04, 1.96 ± 0.07, 1.19 ± 0.04, 1.05 ± , 0.12 and 0.22 ± 0.03 for juveniles (J), males (M), early transitionals (T1), late transitionals (T2) and females (F), respectively. The *AM/AI* ratio was significantly different between life history stages (Kruskal-Wallis, H_(4, 46)_ = 37.61, *P* < 0.01). According to the Tukey’s HSD test, statistical significance (*P* < 0.05) of the *AM/AI* ratio was found between males, females and the rest of the stages (J, T1, T2), while J, T1 and T2 didn’t differ from each other (Fig. 3B).

### Gonad transformation during the life history of *P. platyceros*

**Figure 4:**
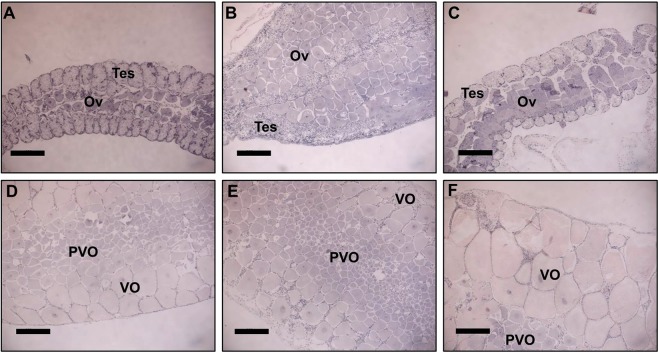
Histological sections stained with hematoxylin and eosin (H&E) from different reproductive stages of *P. platyceros*. (**A**) Juvenile, (**B**) Male, (**C**) Early transitional, (**D**) Late transitional, (**E**) Ovigerous female and (**F**) Non-ovigerous female. Testicular tissue (Tes) and ovarian tissue (Ov) are designated when ovotestis is present. Pre-vitellogenic and vitellogenic oocytes (PVO and VO) are indicated when only an ovary is present. Bars = 0.5 mm.

**Figure 5:**
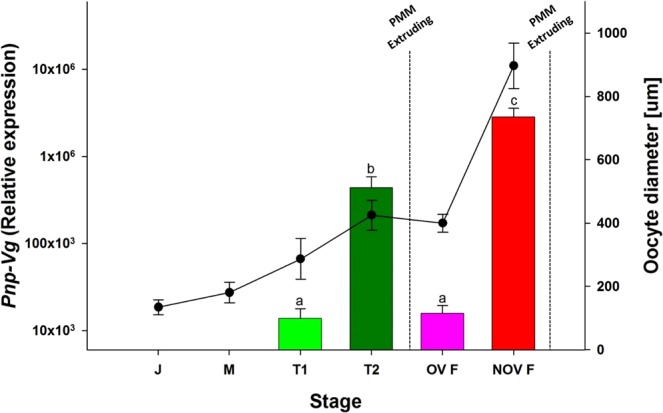
Vitellogenin relative expression (bars) and oocyte diameter (line) in different reproductive stages of *P. platyceros*. (**J**) Juvenile, (**M**) Male, (T1) Early transitional, (T2) Late transitional, (OV F) Ovigerous female and (NOV F) Non-ovigerous female. The stages of the pre-mating molt (PMM) and the time at which eggs are extruded are indicated. Error bars represent standard error of the means and statistical difference is marked with letters.

Histological sections of the gonads (Fig. 4) showed that the ovotestis is present in juvenile, male and early transitional stages. During the late transitional stage (T2) the testicular tissue degenerates and remains degenerated throughout the entire female phase. The oocytes within the gonad increase in size as the animal transforms from maleness to femaleness along the *P. platyceros* life-cycle. More specifically, the average ± SE oocyte diameter measured was 134.32 ± 23.66, 180.65 ± 32.71, 286.72 ± 64.44, 424.75 ± 47.45, 399.73 ± 28.07 and 896.97 ± 71.93 μm for juveniles (J), males (M), early transitionals (T1), late transitionals (T2), ovigerous females (OV F) and non-ovigerous females (NOV F), respectively. Changes in oocyte diameter along the different reproductive phases of *P. platyceros* are presented in Fig. 5 (black curve).

### The vitellogenin gene in *P. platyceros*

**Figure 6:**
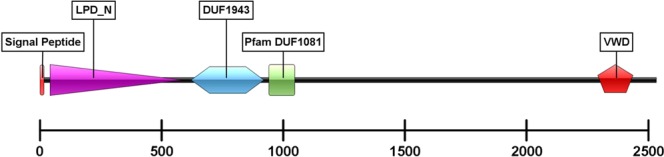
Linear model of the vitellogenin protein in *P. platyceros*. The conserved domains of the protein are indicated: signal peptide, lipoprotein N-terminal domain (LPD_N), two domains of unknown function (DUF) and Von Willebrand factor type D domain (VWD). The location of the amino-acids in the protein is scaled.

**Figure 7:**
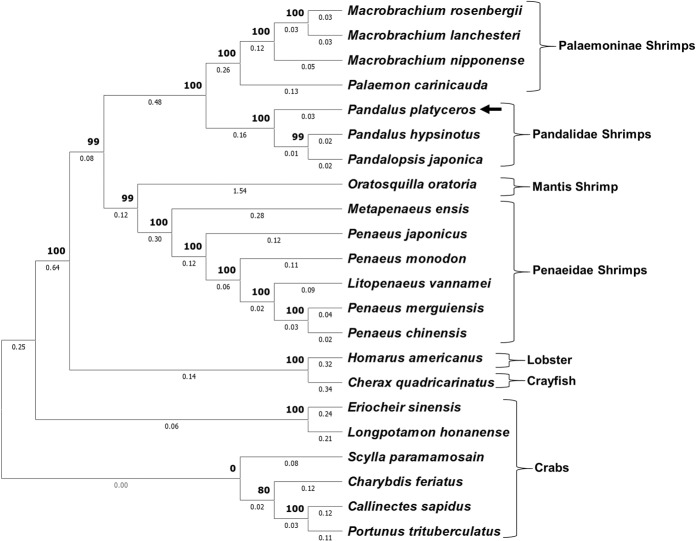
Phylogenetic tree of deduced vitellogenin protein sequences in 22 decapod crustacean species. Pnp-Vg is indicated with a black arrow. Supporting values on junctions (bold) as well as branch lengths are given.

Sequencing the full *Pnp-Vg* mRNA sequence yielded a 7,797 bp sequence which is translated to a 2,544 amino acid putative protein (GenBank accession number: MK070912). The predicted structure of the protein that was inferred from its amino acid sequence (Fig. 6) included a signal peptide (positions 1–18), a lipoprotein N-terminal domain (LPD_N; positions 42–590), a domain of unknown function, DUF 1943 (positions 622–921), a domain of unknown function, Pfam:DUF 1081 (positions 941–1048) and a Von Willebrand factor type D domain (VWD; positions 2289–2439). A phylogenetic tree of Vg amino acid sequences from 22 crustacean decapod species (Fig. 7) showed that Pnp-Vg shares the highest degree of similarity with Vg sequences of other pandalid shrimps.

### Relative expression of Vg throughout the life-cycle of *P. platyceros*

Analysis of the *Pnp-Vg* relative transcript levels revealed that the transcription of *Vg* is significantly different between the different stages (ANOVA, F_(5, 33)_ = 91.05, *P* < 0.05). More specifically, according to the Tukey’s HSD test *Vg* transcription is significantly higher (*P* < 0.05) in animals that are actively undergoing ovarian maturation. Therefore, transcript levels of *Pnp-Vg* measured in juvenile and male shrimps were negligible (Fig. 5 – bars). The levels of *Vg* transcript in the T2 stage was significantly higher (*P* < 0.05) than in the T1 and ovigerous female stages. The levels of *Vg* transcripts increased in females after hatching with transcripts measured in the non-ovigerous females statistically greater than in all other stages (*P* < 0.05).

## Discussion

**Figure 8:**
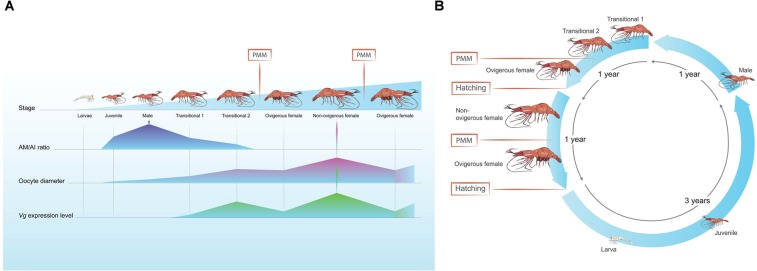
The life cycle of *P. platyceros*. (**A**) Simultaneous representation of the morphological (*AM/AI* ratio) and physiological (oocyte diameter and *Vg* transcript level) changes occurring during the different stages of the life-cycle of *P. platyceros*: larvae, juvenile, male, early transitional (transitional 1), late transitional (transitional 2), ovigerous female and non-ovigerous female. Pre-mating molt (PMM) events are indicated. (**B**) Time scale of the different phases along the life-cycle *P. platyceros*. Times of PMM and hatching are indicated.

The full sequence of *Pnp-Vg* mRNA contains typical conserved domains such as a signal peptide and two other common domains (LPD_N and VWD). Each of these domains are also found within *Vg* genes from diverse species such as other crustaceans^31,32^, insects^33^, fish^34^, frogs^35^ and chickens^36^ which highlights their importance in the function of this yolk protein precursor. The phylogenetic tree of Vg amino acid sequences from 22 decapod species shows that Vg is conserved within specific groups of decapod crustaceans (i.e. crabs, shrimps etc.) and that Pnp-Vg sequence is most similar to the Vg gene of *P. hypsinotus* and *Pandalopsis japonica*, both which are protandric hermaphrodites from the family Pandalidae^25,37–39^. While relative transcript levels of *Pnp-Vg* are negligible in juveniles and males, levels clearly increase as an animal starts the transformation to the functional female physiology. The highest *Vg* transcript levels were found in NOV females and correspond to the accumulation of vitellin and growth of the oocytes in NOV females after hatching. After extruding mature eggs, the *Pnp-Vg* transcript level in ovigerous females is significantly reduced supporting the hypothesis that *P. platyceros* females undergo a single reproductive cycle each year. A single reproductive cycle is also supported by results described in a previous study on the protandric shrimp, *P. hypsinotus*. In that study, Vg protein levels were measured in different stages of females. In March after the eggs hatched, circulating Vg was low and increased between April to October after which the mature eggs were extruded^25^. *Vg* transcript level is a comprehensive transcriptomic parameter used to further describe the different phases along the life-cycle of *P. platyceros* (Fig. 5). However, simultaneous representation of *Vg* transcript level with additional molecular and morphological indicators (Fig. 8A) will clarify the story aiming to explain the protandric nature of this shrimp.

Important life history traits include age at sexual maturity and total life span. Life span can be especially hard to assess in this species but some age estimations have been attempted through mark and recapture methods^11^ and studying the basic biology in field studies^13,40^. A novel and wider approach into the life-cycle of *P. platyceros* is presented in Fig. 8A. This figure illustrates the transcriptomic changes of *Vg* as described above in parallel with the morphological (*AM/AI* ratio) and histological (oocyte diameter) indicators in the different phases of the shrimp’s life-cycle (larval, juvenile, male, early and late transitional and ovigerous and non-ovigerous female).

*AM* and *AI* are prominent characters associated with sex identification in many shrimps and prawns^17,18^. It was previously reported that in the members of the genus *Pandalus*, which are all protandric hermaphrodites, males have fully developed *AM* and *AI* on the second pleopod, transitionals have reduced *AM* and developed *AI* while the *AM* is absent and only an *AI* remains in females^21,37^. Our results are consistent with those described above, but moreover, in our study we examined the *AM/AI* ratio not only in males and females but also in juveniles and two different transitional stages. We can infer that, from post-larval stages to juvenile, the shrimps are developing their *AM* in each subsequent molt and when maturing as a male, the *AM* is already twice the length of the *AI*. Later, the *AM* length is reduced during each transitional molt until it almost completely disappears when a shrimp matures as a female. External observation of the *AM* and *AI* as presented in Fig. 3, and as described in Fig. 8A as a morphological indicator could be used to determine the sexual stage of the animal as a simple method to examine a fishery harvest or when establishing breeding stock populations for mariculture.

Examination of the gonad from each life history stage demonstrates that spot shrimps retain their testicular tissue as part of the ovotestis alongside pre-vitellogenic oocytes until the late transitional stage. At this point, vitellin is accumulating in the maturing oocyte which is concomitantly increasing in size. Once a female extrudes her eggs, most of the remaining oocytes are pre-vitellogenic; thus, are relatively small. Females brood their eggs from late summer until early spring^2^, which implies that only a single reproductive cycle of vitellogenesis is possible for each female annually. This might explain why the oocytes that were not extruded remain small as they will not accumulate vitellin until the next reproductive cycle. Moreover, monitoring oocyte diameter during the life history of this shrimp shows that while the size of the oocyte is increasing from juvenility through maleness and transitional stages to femaleness, the most significant increase in size occurs prior to egg extrusion. This maturation process reflects the important act of accumulating the yolk proteins to provide for the embryos during embryogenesis. Our results are consistent with a previous study conducted on the protandric shrimp *P. hypsinotus* which maintained testicular tissue as part of the ovotestis until a transitional stage after which the testicular tissue degenerated, the oocytes began accumulating yolk protein and circulating hemolymph Vg levels increased^38^.

All the above morphological (*AM/AI*), histological (oocyte diameter) and transcriptomic (*Vg* level) indicators shown simultaneously in Fig. 8A contribute to understand the complex cycle of events each shrimp must go through along its protandric life-cycle.

Northern spot shrimps are important for fisheries along the west coast of North America including Alaska, Canada, Washington, Oregon and California^41^. In Alaska, Northern spot shrimps are an important species for local communities for both recreational and commercial fisheries. Shrimp populations are monitored annually by the ADFG and the annual data is publicly available^3,42^. During the annual survey, shrimps are measured, weighed and sexed. While imposing size restrictions is important to retain juveniles and let them reach maturation to preserve future breeding and spawning^43^, this is even more important in species experiencing a long period between hatching to adulthood. The long period in *P. platyceros* is demonstrated in Fig. 8B, in which females extrude their eggs in the fall and larvae hatch in the spring after approximately 5–7 months, sometime between late March and late May^2^. Berkeley (1930;^9^) reported that 6 larval stages exist, while Price and Chew (1972;^44^) reported 5 larval stages considering the fifth to be actually the first post-larval stage which occurs approximately 40 days post-hatching. At this stage, the benthic juvenile period begins and lasts for at least 2 years until the juvenile matures into the male phase^11,13^. The duration of the male phase within warmer locations of the Pacific Ocean other than Alaska is reported to be approximately a year, and a summer mating is followed by the transformation of the male into the female phase and occurs in the spring. In warmer waters, the maturation of males into a mature female that is ready to mate will occur in late summer at an age of approximately 3–3.5 years^14–16^. In Alaska, it was reported that some females mature at an age of over 5 years^11^. With an estimated time of approximately 5 years from hatching to the spawning ovigerous female phase (Fig. 8B), size restrictions to retain juveniles, males and transitionals seem necessary to protect populations of the protandric spot shrimp. On the other hand, its protandric nature causes distinct sexual size dimorphism with females larger than males. In this case, as in other species with sexual size dimorphism, size regulation of fishery may result in sex-selective fishing that might cause skewed sex-ratio resulting in lower reproductive success^45–47^.

Northern spot shrimp populations in Alaska have experienced declines over the past 25 years and despite fisheries restrictions, populations of shrimps have not been restored to their historic levels. In some districts within the Southeast Alaska management area, shrimp population estimates have led to the temporal closure of the fishery to commercial and personal use harvest. The GHL is determined based on survey data by the ADFG prior to the opening of the fishery. The annual harvest of spot shrimps from 2003 to 2017 correlated well with the established quota permitted by the ADFG. Nevertheless, closures of districts 12, 14, 15 and 16 (Fig. 1) reflect the persistent diminished numbers of spot shrimp stocks in those areas^3^. Given the long period of time required for a spot shrimp to reach female maturity, and given that once mature, only a single annual reproductive event per female (Fig. 8B), temporal closures, even for several years, may not be sufficient to sustain spot shrimp populations. On the other hand, areas that are currently closed to fishing could be designated as permanent marine reserves and could serve as source breeding populations^48^. In a marine reserve, the stock will be protected and since most marine species include a pelagic larval phase in their life cycle, they would be free to hatch and migrate from the protected area to replenish populations in adjacent fishing areas^48^. Moreover, it was reported that even adults and juveniles are emigrating beyond the reserve boundaries when stocks build-up^49^. As a consequence of establishing permanent marine reserves and protecting the spawning stock, populations are enhanced as are catches in adjacent fishing areas^7,48,50,51^.

While gear restrictions are important for managing the target fishery, they also serve to minimize the destructive effects of particular gear on the marine ecosystem as well as reducing bycatch of non-target species^52^. For instance, there is a trawl fishery for shrimps that is regulated to allow for catch of target species^3^. However, trawl fisheries also can result in severe damage to marine habitats^53,54^ and to increase in the by-catch of non-target species populations, such as marine mammals, turtles, birds, fish and other marine invertebrates^55,56^.

While monitoring shrimp populations is of importance for managing fisheries, pandalid shrimps were also suggested to be important for ecological concepts. A previous study showed that ecosystem health can be evaluated through the monitoring of shrimps and that pandalid shrimp populations may serve as indicators for climate change^57^. Shrimp populations within the Gulf of Alaska experienced a rapid decline after 1977 which was associated with an abrupt warming of the water column.

To summarize, Northern spot shrimps are not only important for commercial, subsistence and recreational harvest but also for ecological considerations. As is evident from the schematic presentation in Fig. 8, the spot shrimp life-cycle and reproductive physiology shows a longer time frame from hatching to maturation than in previous studies. It is now described that females require at least 4 years to maturation and that each reproductive cycle requires at least a year. Current regulations and management of the protandric spot shrimp may require a more conservative approach. Temporary fishery closures for several years may not be sufficient to enable viable consecutive generations of shrimps. Moreover, closures will not be sufficient in areas where prior trawl fishing was allowed since even if regulated, it may take decades for the marine ecosystem to recover from its destructive effects. We believe that the solution to sustaining shrimp populations in Alaska and elsewhere is permanent marine sanctuaries, which are permanently closed for fishing, will keep the protected stock healthy and will most likely increase catches in adjacent fishing areas. It is noteworthy that in a protandric species with sexual size dimorphism, a standard fisheries management measure such as size regulations may result in sex selective harvest and therefore, even more environmentally friendly gear which is used in pot fishery may cause reduction in reproductive success, which might lead to rapid declines of shrimp populations. We believe that this thorough molecular study of the reproductive physiology of this species was necessary for shedding new light on the biology of this commercially important protandric species and thus will provide better management information for commercial, subsistence and recreational shrimp fisheries.

## Methods

### Shrimp pot fishery data analysis

We conducted a meta-analysis of shrimp pot fishery data obtained from the Alaska Department of Fish and Game (ADFG)^3^ to assess changes in *P. platyceros* populations within Southeast Alaska over the past fourteen years. Commercial shrimp harvest data was obtained from twelve of the sixteen defined management regions in Southeast Alaska (Fig. 1). Guideline harvest levels (GHL) are determined annually from annual pot survey data and inform shrimp stock assessments. Harvest data were not available for 4 of the districts (districts 4, 5, 14 and 16) due to closures so data was analyzed from the twelve fished districts. Harvest data and GHL from 12 management districts were combined for all years from 2003 to 2017. Additionally, actual harvest levels in all districts except four (4, 5, 14 and 16) from 1993 to 2017 were combined and averaged. Harvest data and GHL determined for a representative location (district 1, Fig. 1) were specifically analyzed to determine changes in shrimp harvest in the area where the animals for the reproductive physiology part of this study were collected.

### Animals and life stage definition

*P. platyceros* animals were collected in Behm Canal, Southeast Alaska (management district 1 – Fig. 1) using baited pots during the annual shrimp survey conducted by the ADFG during September 2017. The animals were transferred to the University of Alaska Southeast (UAS) marine laboratory in Juneau and maintained in 750-L tanks in flow-through seawater with ambient light and temperature throughout the year. The animals were fed *ad libitum* (herring chunks, squid or clams). In *P. platyceros*, the *AM* located on the second pleopod develops as the juvenile shrimp matures to a male, following gradual decrement in length as the shrimp fully transforms into a female^21^. Therefore, the reproductive stage of each animal was determined using morphological parameters (i.e. using *AM/AI* ratio, as an indicator^21^). Juveniles (J) were small animals (total body length of 4 to 8 cm) with *AM/AI* ≤ 1. Mature males (M) were medium sized animals (total body length of 12 to 16 cm) *AM/AI* ≈ 2. The transitional phase during which the male transforms into the female was separated into two stages based on the *AM/AI* ratio: Early transitional (T1), represented the first transitional stage and occurred one molt after the M phase (the first molt in which the *AM* becomes shorter), with *AM/AI* ≈ 1. Late transitional (T2), is the phase that occurs one molt after the T1 stage and the AM/AI is < 1 The female phase was separated into two stages: Ovigerous female (OV F): a female with extruded eggs, and a non-ovigerous female (NOV F): a post hatch female (over three months since hatching) with a well-developed ovary (visually determined) that is preparing for PMM.

### External characteristics of sexual stage in *P. platyceros*

As previously described^21^, The *AM* and *AI* measurements can be used to determine the sexual reproductive stage of *P. platyceros*. In order to follow external characters of the shrimp associated with the different stages along its life-cycle, second pleopods were removed from each life history stage of *P. platyceros*: juveniles (n = 8), males (n = 9), early transitionals (n = 12), late transitionals (n = 7) and females (n = 10). The *AM* and *AI* were measured using ImageJ software^58^, and the *AM/AI* ratio was compared between the different stages.

### Histology and oocyte measurements

In order to examine morphological changes of the gonads during the life-cycle of *P. platyceros* from juvenility to femaleness, three animals from each stage (J, M, T1, T2, OV F and NOV F) were randomly sampled and their gonad was dissected and fixed for histology as previously described^19^. Fixed gonads were gradually dehydrated through a series of increasing alcohol concentrations, incubated with xylene, and embedded in Paraplast (Kendall, Mansfield, MA, USA). Consecutive sections of 5 μm were placed on silane-coated slides (Menzel-Gläser, Braunschweig, Germany) and stained with hematoxylin and eosin (H&E) for morphological observations as previously described^59^. For each gonad, the diameter of representative oocytes (n = 3) was measured using ImageJ software^58^ and oocyte size of each of the different stages was compared. To confirm consistency of the measured area of the oocyte between different slides, the diameter was measured only in oocytes where the nucleus was visible.

### The vitellogenin gene in *P. platyceros*

Total RNA was extracted from the hepatopancreas of a *P. platyceros* female using EZ-RNA Isolation kit (BI, Cromwell, CT, USA), and cDNA was prepared using qScript cDNA Synthesis kit (Quanta, Beverly, MA, USA) according to the manufacturer’s protocols. Based on the vitellogenin mRNA of *P. hypsinotus* (GenBank accession number: AB117524.1), forward (5′-TGGTGAGATGGGCAATGACTGGATGA-3′) and reverse (5′-GCACTGCTGATCTTCCTGCCACGAT-3′) primers were designed. The vitellogenin mRNA in *P. platyceros* (*Pnp-Vg*), was obtained by the rapid amplification of cDNA ends (RACE) method using SMARTer RACE cDNA Amplification kit (Clontech, Mountain View, CA, USA), with the above-mentioned primers and the Universal Primers Mix (UPM) from the RACE kit (including the long universal primer: 5′-CTAATACGACTCACTATAGGGCAAGCAGTGGTATCAACGCAGAGT-3′, and the short universal primer: 5′-CTAATACGACTCACTATAGGGC-3′). Additional PCR amplifications of the 5′ and 3′ regions were performed using specific primers (3RACE_For: GCCAGACTCCCAGTGTATCCCCTGCTAG ′, 5RACE_Rev: 5′-TCCCAGGAGACCGGCCAATTGACCAAAG-3′) and the UPM with the above mentioned RACE kit. After sequencing the entire *Pnp-Vg* mRNA, the predicted domains of the putative protein were inferred from its deduced amino acids sequence using SMART (http://SMART.embl-heidelberg.de;)^60^. In addition, multiple sequence alignment (MSA) of Pnp-Vg with Vg peptides from 22 different decapod crustacean species (Table S1) was performed using MAFFT program version 7^61^. In order to test what is the best model to select with likelihood-based criteria we used the Smart Model Selection (SMS) function with Bayesian information criterion (BIC) in PhyML^62^. The fittest chosen model that was eventually performed is JTT^63^ and model decoration (i.e. RAS and equilibrium frequency options) were + G + I + F^62^. The evolutionary phylogenetic analysis was visualized using MEGA X^64^.

### *In-vitro* expression of *Pnp-Vg* transcript in different stages

We hypothesized that *Pnp-Vg* transcript levels will increase during transformation from maleness to femaleness and will reach a peak in the NOV stage, just before eggs extrusion. In order to test it, total RNA was extracted and cDNA was synthetized, as described above, from the hepatopancreas of *P. platyceros* animals at different stages: J (n = 6), M (n = 4), T1 (n = 8), T2 (n = 7), OV F (n = 8) and NOV F (n = 6). Additionally, RNA was extracted from the androgenic gland (AG) of a mature male as a control to normalize the qPCR assay. Relative quantification of transcript levels was performed using Roche Diagnostics FastStart Universal Probe Master Mix (Basel, Switzerland) and Roche Universal Probe Library probes. The following primers and probe were used: qPnp-Vg F (5′-TGTGCAACTAAGGGAGTTATGGA-3′) and qPnp-Vg R (5′-GGTGAGTGCCAAAGAAGAGTG-3′), and Probe #89. *P. platyceros* 18 S, which served for normalization, was also quantified by means of qPCR using the primers, qPnp-18S F (5′-CCCTAAACGATGCTGACTAGC-3′) and qPnp-18S R (5′-TACCCCCGGAACTCAAAGA-3′), and Probe #152. Reactions were performed in the ABI Prism7300 Sequence Detection System, Applied Biosystems (Foster City, CA).

### Statistical analysis

In order to test fitness for proper statistical analysis, all data was first tested for residuals normality using the Shapiro-Wilk test and for homogeneity of variance using the Levene’s test. As previously described^65^, analyzing ratio data sets of groups with unequal sample sizes is recommended to be performed by a non-parametric test. Therefore, statistical difference of the *AM/AI* ratio between the different stages was tested using Kruskal-Wallis test followed by a post-hoc Tukey’s HSD test. For the relative transcript levels of *Pnp-Vg* the data was first logarithmically transformed as to facilitate a proper statistical analysis and then analyzed using one-way ANOVA followed by a post-hoc Tukey’s HSD test. All statistical analyses were performed using Statistica v13.3 software (StatSoft Ltd., Tulsa, OK, USA).

## Supplementary information

Table S1.
