# Supplementary material for: The protandric life history of the Northern spot shrimp Pandalus platyceros: molecular insights and implications for fishery management
Source: Sci Rep. 2020 Jan 28;10:1287. doi: 10.1038/s41598-020-58262-6 (PMC6987223; doi:10.1038/s41598-020-58262-6)
Supplement: Supplementary file 1 — Table S1. [file 41598_2020_58262_MOESM1_ESM.docx]

**The protandric life history of the Northern spot shrimp *Pandalus platyceros*: molecular insights and implications for fishery management**

**Tom Levy^1^, Sherry L. Tamone^2*^, Rivka Manor^1^, Esther D. Bower^2^ and Amir Sagi^1,3*^**

^1^Department of Life Sciences, Ben-Gurion University of the Negev, P.O. Box 653, Beer Sheva 84105, Israel

^2^University of Alaska Southeast, 11066 Auke Lake Way Hwy, Juneau, AK, 99801, USA

^3^The National Institute for Biotechnology in the Negev, Ben-Gurion University of the Negev, P.O. Box 653, Beer Sheva 84105, Israel

*Authors to whom correspondence should be addressed:

Emails: [sltamone@alaska.edu](mailto:sltamone@alaska.edu); [sagia@bgu.ac.il](file:///\\biostorage1\public\Files\sagialab\Rivka\Genes%20silencing%20review\revised%20paper\sagia@bgu.ac.il)

**Supplemental material**

**Table S1: List of 21 known vitellogenin sequences in different decapod crustacean species.** The sequences from the list were used for phylogenetic analysis of the vitellogenin gene. GenBank accession numbers are indicated.

| **Species** | **Accession number** | **Gene name** |
| --- | --- | --- |
| *Callinectes sapidus* | DQ314748.1 | *Callinectes sapidus* vitellogenin mRNA, complete cds |
| *Portunus trituberculatus* | DQ000638.1 | *Portunus trituberculatus* vitellogenin mRNA, complete cds |
| *Scylla paramamosain* | FJ812090.1 | *Scylla paramamosain* vitellogenin mRNA, complete cds |
| *Charybdis feriatus* | AY724676.1 | *Charybdis feriatus* vitellogenin mRNA, complete cds |
| *Longpotamon honanense* | KP319023.1 | *Longpotamon honanense* vitellogenin mRNA, complete cds |
| *Eriocheir sinensis* | KC699915.1 | *Eriocheir sinensis* vitellogenin mRNA, complete cds |
| *Homarus americanus* | EF422415.1 | *Homarus americanus* vitellogenin mRNA, complete cds |
| *Cherax quadricarinatus* | AF306784.1 | *Cherax quadricarinatus* vitellogenin (vg) mRNA, complete cds |
| *Pandalus hypsinotus* | AB117524.1 | *Pandalus hypsinotus* Vg mRNA for vitellogenin, complete cds |
| *Pandalopsis japonica* | ACU51164.1 | vitellogenin [*Pandalopsis japonica*] |
| *Palaemon carinicauda* | AFM82474.1 | vitellogenin [*Palaemon carinicauda*] |
| *Macrobrachium nipponense* | KJ768657.1 | *Macrobrachium nipponense* vitellogenin mRNA, complete cds |
| *Macrobrachium rosenbergii* | AB056458.1 | *Macrobrachium rosenbergii* Macr-Vg mRNA for vitellogenin, complete cds |
| *Macrobrachium lanchesteri* | AQX37249.1 | vitellogenin [*Macrobrachium lanchesteri*] |
| *Metapenaeus ensis* | AF548364.1 | *Metapenaeus ensis* vitellogenin mRNA, complete cds |
| *Penaeus japonicus* | BAD98732.1 | vitellogenin [*Penaeus japonicus*] |
| *Penaeus monodon* | DQ288843.1 | *Penaeus monodon* vitellogenin mRNA, complete cds |
| *Litopenaeus vannamei* | AY321153.2 | *Litopenaeus vannamei* vitellogenin (Vg) mRNA, complete cds |
| *Penaeus merguiensis* | ACV32381.1 | vitellogenin [*Penaeus merguiensis*] |
| *Penaeus chinensis* | ABC86571.1 | vitellogenin [*Penaeus chinensis*] |
| *Oratosquilla oratoria* | ALI16501.1 | vitellogenin [*Oratosquilla oratoria*] |
